# Oxo-Titanium(IV) Complex/Polymer Composites—Synthesis, Spectroscopic Characterization and Antimicrobial Activity Test

**DOI:** 10.3390/ijms21249663

**Published:** 2020-12-18

**Authors:** Piotr Piszczek, Barbara Kubiak, Patrycja Golińska, Aleksandra Radtke

**Affiliations:** 1 Faculty of Chemistry, Nicolaus Copernicus University in Toruń, Gagarina 7, 87-100 Toruń, Poland; basiak0809@gmail.com; 2 Faculty of Biological and Veterinary Sciences, Nicolaus Copernicus University in Toruń, Lwowska 1, 87-100 Toruń, Poland; golinska@umk.pl

**Keywords:** antimicrobial activity, oxo-titanium(IV) complexes, polymer-inorganic composites, physicochemical properties, thermal properties

## Abstract

The emergence of a large number of bacterial strains resistant to many drugs or disinfectants currently used contributed to the search of new, more effective antimicrobial agents. In the presented paper, we assessed the microbiocidal activity of tri- and tetranuclear oxo-titanium(IV) complexes (TOCs), which were dispersed in the poly(methyl methacrylate) (PMMA) matrix. The TOCs were synthesized in reaction to Ti(OR)_4_ (R = ^i^Pr, ^i^Bu) and HO_2_CR’ (R’ = 4-PhNH_2_ and 4-PhOH) in a 4:1 molar ratio at room temperature and in Ar atmosphere. The structure of isolated oxo-complexes was confirmed by IR and Raman spectroscopy and mass spectrometry. The antimicrobial activity of the produced composites (PMMA + TOCs) was estimated against Gram-positive (*Staphylococcus aureus* ATCC 6538 and *S. aureus* ATCC 25923) and Gram-negative (*Escherichia coli* ATCC 8739 and *E. coli* ATCC 25922) bacteria and yeasts of *Candida albicans* ATCC 10231. All produced composites showed biocidal activity against the bacteria. Composites containing {Ti_4_O_2_} cores and the {Ti_3_O} core stabilized by the 4-hydroxybenzoic ligand showed also high activity against yeasts. The results of investigations carried out suggest that produced (PMMA + TOCs) composites, due to their microbiocidal activity, could find an application in the elimination of microbial contaminations in various fields of our lives.

## 1. Introduction

The unique physical and chemical properties of materials based on titanium dioxide, i.e., photocatalytic activity, hydrophilicity, or strong absorption of UV radiation, contribute to their wide-application inter alia in various fields of chemical, cosmetics, biomedical, pharmaceutical, and disinfecting applications [1,2,3,4,5]. The photocatalytic activity of TiO_2_ nanoparticles or nanocoatings, associated with the electron transfer between the valence band and the conduction one (the O2*p*-Ti3*d* charge transfer transition), which can take place in the presence of photons of wavelengths smaller than 350 nm (this corresponds to ca. 5% of the daylight), is of particular importance [3,4,6]. The above-mentioned phenomenon initiates the oxidation and reduction reactions, which proceed on the surface of TiO_2_ nanoparticles/nanocoatings and lead to the formation of the reactive oxygen species (ROS) responsible for the antimicrobial activity of these materials, among others [3]. Aiming to extend the absorption range of TiO_2_ in daylight, attention was paid to the oxo-titanium(IV) complexes (TOCs), which contain {Ti_a_O_b_} cores of different architectures in their structures and their potential use as antimicrobial and photocatalytic active substrates [7,8,9,10,11]. These compounds are studied for the possibility of their use for the photoinduced degradation of organic dyes or water splitting [8,12]. In the case of TOCs, the electron transfer takes place also between the 2*p* electron shell of the oxygen atom and the 3*d* shell of the titanium atom; however, the presence of ligands, which stabilize the oxo-titanium(IV) complex cluster, act as photosensitizers, and in this way, the radiation can be absorbed in a wider range [13,14]. By placing the ligands in the coordination sphere (e.g., carboxylate ligands (-O_2_CR’)), the orbitals HOMO-LUMO of ligands and the {Ti_a_O_b_} core can be mixed. As a result, the distance for electrons to move is smaller. The charge transfer from the ligands to the cluster core is possible due to its low-energy absorption band [15,16]. The photocatalytic activity of the oxo-titanium(IV) complexes, of the different {Ti_a_O_b_} core architectures and possessing various carboxylic ligands, was most often assessed based on the photodegradation processes of organic dye aqueous solutions, such as methylene orange (MO), methylene blue (MB), and rhodamine B (RB) [16,17,18,19]. The samples, during the photocatalytic experiments were mostly irradiated by UV light in the range 315–400 nm; however, also, visible light was used. Kim et al. studied the oxo-Ti(IV) complexes with the {Ti_6_O_6_} core, and stabilized by such carboxylate ligands as 4-aminobenzoic, 4-amino-2-fluoro-benzoic, 4-amino-2-chloro-benzoic, 4-amino-3-chloro-benzoic, and 5-dichlorobenzoic acids, the anions were especially interesting. They showed a direct dependence of oxo-complex photocatalytic activity versus the way of -O_2_CR’ group functionalization. This effect is closely related to the change in the distance between HOMO and LUMO orbitals in the tested compounds [19]. The results of our earlier studies on the photocatalytic activity of oxo-clusters with {Ti_3_O} and {Ti_4_O_2_} cores also exhibited a significant influence in the way carboxylate ligands functionalize [20,21,22]. According to these works, the oxo-Ti(IV) complexes stabilized by 9-fluorenecarboxylate and 4-aminobenzonate ligands revealed the best photocatalytic properties in the photodegradation of the methylene blue (MB) solution [20,21].

An interesting issue that may be related to the photocatalytic activity of oxo-Ti(IV) complexes are their antimicrobial properties. Nowadays, searching for new antimicrobial materials results from the necessity of their wide use in everyday life and from their importance in the public health system. Analyzing the literature data, we turned our attention on the antibacterial activity of compounds that contain metal-oxo cores, e.g., oxo-iron(III) complexes [23] or polyoxometalates [24], in their structures. The antibacterial assay of the (Fe_3_O(PhCO_2_)_6_(MeOH)_3_)(NO_3_)(MeOH)_2_ cluster showed a significant growth inhibition of *Bacillus cereus* MTCC 1272, *Staphylococcus epidermidis* MTCC 3086, and *Salmonella typhimurium* MTCC 98 but not of *Escherichia coli* MTCC 723 [23]. The results of studies on the antibacterial properties of polyoxometalates against *Moraxella catarrhalis* seem to be particularly interesting. It has been revealed that microbiocidal activity of these types of compounds mainly depends on their composition; metal-oxide anion core shape and size; and the type of the central metal in MO_6_ unit (M = Mo, V, and W) [24]. Antimicrobial properties have been found in the mixed ligand titanium dioxide complex, which was produced from TiO_2_ nanoparticles and 8-hydroxyquinoline and glycine as the ligands [25]. This complex showed good antifungal activity against *Alternaria alternata*, *Rhizoctania solani*, *Helminthosporium oryzae*, *Fusarium oxysporum*, *Curvularia lunata*, and *Aspergillus niger*, as well as antibacterial activity against *Salmonella*, *E. coli*, *S. epidermis*, and *Enterococcus faecalis*. However, this compound did not show biocidal activity against *Aspergillus fumigatus*, *Aspergillus terreus*, *Trichoderma viride*, and *Cladosporium herbarium* [25]. The investigations of Kaushal et al. revealed the promising biological activities of titanium complexes synthesized by reacting to TiCl_4_ with Schiff bases (SBs) [26,27]. The synthesized complexes were tested for their antimicrobial activity against pathogenic bacterial strains i.e., *B. cereus* MTCC 6728, *Micrococcus luteus* MTCC 1809, *S. aureus* MTCC 3160, *S. epidermidis* MTCC 3086, *Aeromonas hydrophila* MTCC 1739, *Aclaligenes faecalis* MTCC 126, *Shigella sonnei* MTCC 2957, *Klebsiella pneumoniae* MTCC 3384, *Pseudomonas aeruginosa* MTCC 1035, and *S. typhimurium* MTCC 1253. The (TiCl_2_(SB)_2_) complexes showed higher antimicrobial activity than their parent Schiff bases [27]. The analysis of the literature reports have shown that the antimicrobial activity of multinuclear oxo-titanium(IV) complexes have not been fully explored so far. Attention should be drawn to the results of the study by Svensson et al., which revealed the antibacterial activity of the oxo-complexes that consisted of {Ti_4_O_2_} cores stabilized by two triclosan ligands against *S. aureus* [28]. However, in this case, the antibacterial activity of the hydrolyzed form of synthesized complex was studied by the authors.

It should be noted that all the above-mentioned antimicrobial agents can be used in liquid form, which distinguishes them from TOCs systems, which are discussed in the presented paper. The studied compounds (TOCs) containing {Ti_3_O} and {Ti_4_O_2_} cores due to their hydrophobic natures and their resistance to hydrolysis processes [20,21,22] were dispersed in a poly(methyl methacrylate) (PMMA) matrix (i.e., films (PMMA + TOCs) were produced). The aim of our investigations was the synthesis of a (PMMA + TOCs) composite characterized by very high surface antimicrobial activity. PMMA is an important material, which, due to sufficient mechanical properties and minimal inflammatory response, is used in different fields of our lives—inter alia, in dentistry [29]. An important direction of the research related to the use of PMMA-based materials is their modification aimed to improving their antimicrobial activity. This effect can be achieved by the addition of inorganic agents such as silver nanoparticles, titanium dioxide, a mixture of titanium dioxide and silicon dioxide nanoparticles, and anion powder (Na_2_SiO_3_) [29,30,31,32,33,34]. In this paper, the results of the modification of the PMMA films by the introduction of TOC micro-grains are discussed. The influence of TOC structures on the physicochemical properties and photocatalytic and microbicidal activity of the PMMA + TOC systems was assessed.

## 2. Results

Studied oxo-complexes (TOCs) were isolated from a the reaction mixture of appropriate titanium alkoxides and organic acids (4:1 alkoxide: acid molar ratio) at room temperature (RT) and at an inert gas atmosphere (Ar, Schlenk line), according to earlier described procedures (Table 1) [20,21,22]. 4-aminobenzoic acid (HOOC-4-PhNH_2_) and 4-hydroxobenzoic acid (HOOC-4-PhOH) were used in these reactions. The type of the titanium alkoxide (OR; R = ^i^Pr and ^i^Bu) is the factor that directly influences the {Ti_a_O_b_} core architecture.

**Figure 1:**
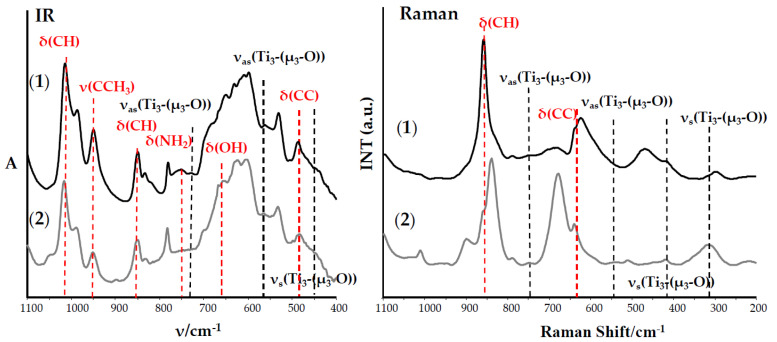
IR and Raman spectra of the (**1**) and (**2**) complexes.

**Figure 2:**
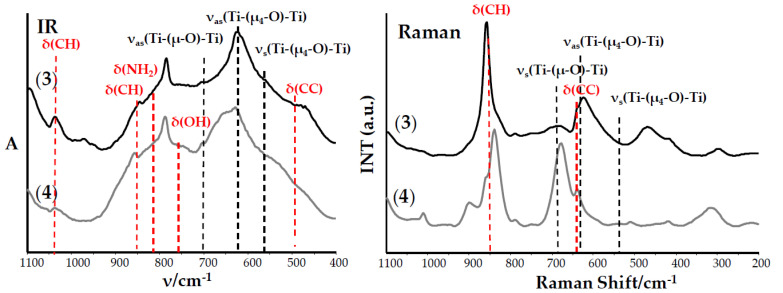
IR and Raman spectra of the (**3**) and (**4**) complexes.

Unfortunately, the weak quality of the formed crystals that caused the structure of isolated solid reaction products were determined based on the analysis of IR, Raman spectra (Figure 1 and Figure 2 and Table 2), and mass spectrometry ones (Table 3).

The vibrational spectra analysis of the (**1**)–(**4**) compounds (Table 2) confirmed the presence of the coordinated carboxylate ligands 1514–1536 cm^−1^ ν_as_(COO)) containing 1,4-substituted Ph groups 780–784 cm^−1^ (ν(CH), IR spectra) functionalized by NH_2_ or OH groups (3100–3500 cm^−1^) and, also, alkoxide groups 1014–1036 cm^−1^ and 948–988 cm^−1^ (ν(Ti-OR)) in their structures. The identification of normal vibrations bands, which descended from titanium-oxide bridge modes, was the spectral proof of the formed {Ti_a_O_b_} core type. For this purpose, the results of our previous DFT calculations were used [20,21,22]. The medium bands at 483 and 483 cm^−1^ in the IR spectra of (**1**) and (**2**) and weak and very weak bands, which were found at 719–728 cm^−1^, 539–564 cm^−1^, and 342–419 cm^−1^ in the IR and Raman spectra of these compounds, were attributed to vibrations of {Ti_3_-(μ_3_-O)} cores (Figure 1 and Table 2).

Analysis of the electrospray ionization-mass spectrometry (ESI-MS) spectra of (**1**) and (**2**) confirm the presence of peaks, which can be assigned to the fragmentation ions containing {Ti_3_-(μ_3_-O)} cores (Table 3). Considering these data, we can state that the trinuclear oxo-Ti(IV) complexes, i.e., [Ti_3_O(O^i^Pr)_8_(O_2_C-4-PhNH_2_)_2_] (**1**) and [Ti_3_O(O^i^Pr)_8_(O_2_C-4-PhOH)_2_] (**2**) were isolated from the 4:1 reaction mixture of Ti(O^i^Pr)_4_ and HO_2_CR’; R’ = 4-PhNH_2_ and 4-PhOH using the tetrahydrofuran (THF)/Pr^i^OH (1:1 mixture) as a solvent. The bands, which were detected at 702 and 703 cm^−1^ and 687 and 690 cm^−1^ in the IR and Raman spectra of (**3**) and (**4**), were attributed to stretching modes of Ti_4_(μ_4_-O) bridges, whereas bands at 634–639 cm^−1^ and 532–556 cm^−1^ (IR) and 687–690 cm^−1^ and 536–548 cm^−1^ (Raman) were assigned to vibrations of Ti_4_-(μ-O) bridges of {Ti_4_O_2_} cores (Figure 2 and Table 2). Additionally, in these cases, the presence of peaks attributed to fragmentation ions containing {Ti_4_O_2_} cores in MS spectra of (**3**) and (**4**) confirms the tetranuclear structures of these complexes (Table 2). The results obtained indicate the isolation of the tetranuclear Ti(IV) oxo-complexes [Ti_4_O_2_(O^i^Bu)_10_(O_2_C-4-PhNH_2_)_2_] (**3**) and [Ti_4_O_2_(O^i^Bu)_10_(O_2_C-4-PhOH)_2_] (**4**) from the mother liquor composed of a 4:1 mixture of Ti(O^i^Bu)_4_ and the above-mentioned organic acids and a 1:1 mixture of THF and Bu^i^OH as a solvent.

### 2.1. UV–Vis Diffuse Reflectance Spectra (UV-Vis-DRS) of the (**1**)–(**4**) Oxo-Complexes and HOMO-LUMO Gap Determination

**Figure 3:**
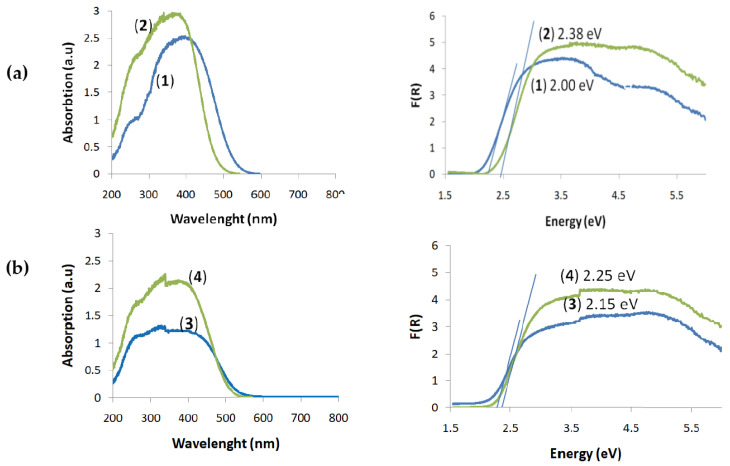
Solid-state UV-Vis-diffuse reflectance spectra (DRS) of the (**1**)–(**4**) micro-grains (the left side) and Kubelka-Munk function versus light energy plot for the HOMO-LUMO gap determination (the right side): (**a**) [Ti_3_O(O^i^Pr)_8_(O_2_CC_6_H_4_NH_2_)_2_] (**1**) and [Ti_3_O(O^i^Pr)_8_(O_2_CC_6_H_4_OH)_2_] (**2**) and (**b**) [Ti_4_O_2_(O^i^Bu)_10_(O_2_CC_6_H_4_NH_2_)_2_] (**3**) and [Ti_4_O_2_(O^i^Bu)_10_(O_2_CC_6_H_4_OH)_2_] (**4**).

UV-Vis-DRS spectra of the (**1**)–(**4**) oxo-complexes were registered at room temperature using magnesium oxide as a standard reference (Figure 3). The HOMO-LUMO gap values were determined basing on the Kubelka-Munk (K-M) function versus light energy, i.e., K = f(*hν*), where K = (1−R)^2/^2R and R is the reflectance, which was used for the optical band gap determination (Figure 3). According to these data, trinuclear oxo-complexes (**1**) and (**2**) exhibit absorption on the border of the UV and Vis range, λ_max_ = 400 nm for (**1**) and λ_max_ = 360 nm for (**2**), and with a sharp absorption edge at ca. 520 nm and 480 nm for complexes (**1**) and (**2**), respectively (Figure 3a). The wide absorption bands with maxima at 405 nm and 390 nm and with the same sharp absorption edge at 500 nm were found in the spectra of (**3**) and (**4**), respectively (Figure 3b). The determined HOMO-LUMO energy gap values (Δ*E*) revealed significant differences between the oxo-complexes with a different type of stabilized carboxylate groups. The lowest Δ*E* values were found for (**1**), Δ*E* = 2.0 eV, and (**3**), Δ*E* = 2.15 eV, i.e., oxo-complexes containing -O_2_C-4PhNH_2_ ligands. The HOMO-LUMO energy gap values of complexes that contain the -O_2_C-4-PhOH groups in their structures were the highest and were equal to Δ*E* = 2.38 eV for (**2**) and Δ*E* = 2.25 eV for (**4**) (Figure 3).

### 2.2. (PMMA + TOC) Composites

**Figure 4:**
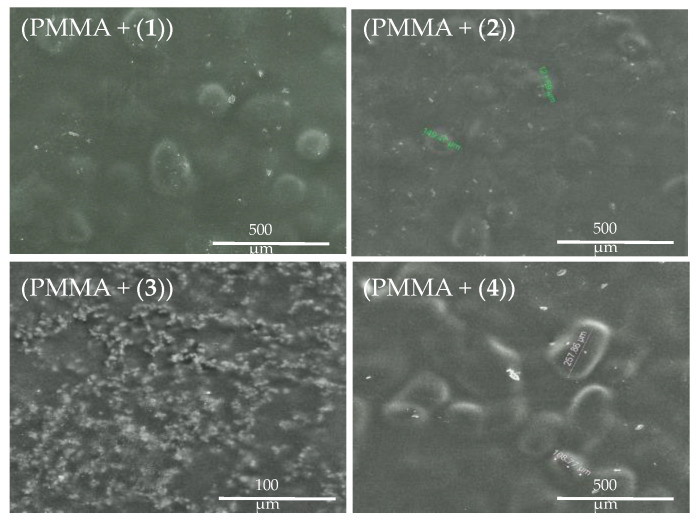
SEM images of poly(methyl methacrylate) (PMMA) + TOCs and TOCs= (**1**)–(**4**) composites dispersed in the PMMA matrix.

**Figure 5:**
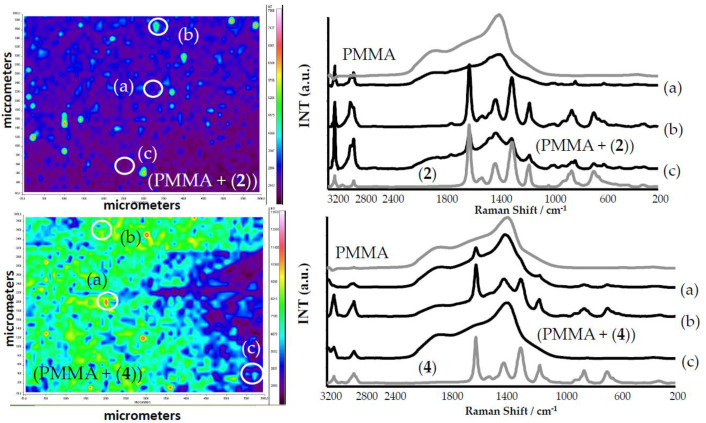
Raman microscopy maps, registered for PMMA + TOC composites (TOCs (**2**) and (**4**)). The Raman spectra, in selected points (a)–(c) on the surface of PMMA + TOCs, are presented alongside.

The photocatalytic properties of the (**1**)–(**4**) titanium(IV) oxo-complexes (TOCs) and their antimicrobial activity were estimated using (PMMA + TOC) composite foils produced by the dispersion of 20 wt.% TOC micro-grains in the PMMA matrix [21,22]. SEM images, presented in Figure 4, show that composite foils (PMMA + (**1**), (**2**), and (**4**)) contain the dispersed TOC grains of diameters 100–300 μm. In the case of (PMMA + (**3)**), fine grains (of diameters 3–5 μm) of the oxo-complex that may form larger aggregates are dispersed in the polymer matrix. The registration of Raman microscopy maps allowed to investigate the TOC grain presence and distribution in the PMMA matrix (Figure 5).

An analysis of the Raman spectra of composite foils proved the presence of tri- and tetranuclear titanium(IV) oxo-complexes of the earlier determined structure. The use of an ultrasonic bath for the dispersion of TOC grains in the polymer solution allowed the appropriate uniform distribution of the fine grains of TOCs throughout the entire polymer volume (Figure 5). However, probably, the solvent evaporation led to the formation of larger TOC grains and places dominated by the polymer.

### 2.3. Thermal Analysis of PMMA/TOC Composites

**Figure 6:**
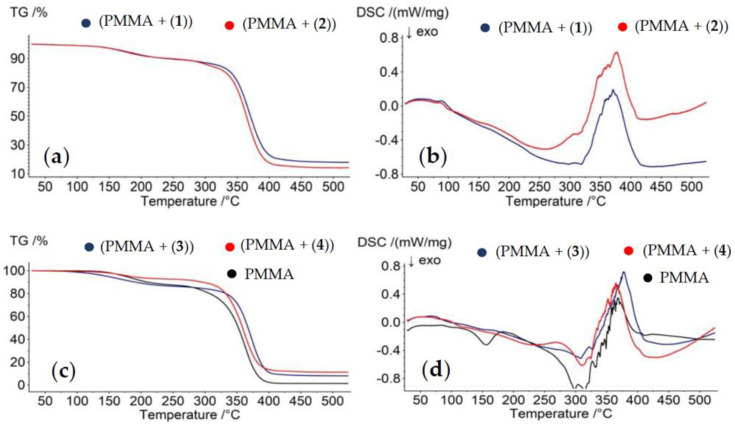
Thermogravimetric curves (TGA) (**a**,**c**) and the differential scanning calorimetry curves of (DSC) (**b**,**d**) of the produced composite materials (PMMA + (**1**)–(**4**)) and PMMA.

The use of thermogravimetric analysis (TGA) and differential scanning calorimetry (DSC) allowed the thermal characterization of (PMMA + TOCs) composites and the PMMA matrix in the temperature range 30–500 °C at inert atmosphere. The results of these investigations are presented in Table 4 and Figure 6. The analysis of the TGA data of all studied samples revealed a slight weight loss (10–15%), which occurred below 280 °C (I Stage), and the major one (70–85%) between 280 and 450 °C (II Stage). Considering the previous investigations, Stage I can be attributed to the loss of the residual solvent and/or monomer, while Stage II was due the samples’ thermal decomposition [35,36].

An analysis of the DSC thermograms exhibits that the PMMA matrix has a glass transition temperature (T_g_) at 99.6 °C, while the exothermic region at 150.8 °C (T), according to previous reports, can be assigned to the evaporation of an unreacted monomer [37] (Figure 6d). The latter effect in the DSC curves of (PMMA + (**1**)–(**4**)) samples was not observed. The endothermic peak at 368.6 °C, which was found on the PMMA DSC thermogram, can be assigned to the PMMA melting temperature (T_m_). In the case of composite films enriched with (**1**), (**2**), and (**3**) oxo-complexes, the strong endothermic peaks appear at 370.4 °C, 375.7 °C, and 377.8 °C; this is above the T_m_ of the pure PMMA matrix. The exception is (PMMA + (**4**)), for which the T_m_ was registered at about 364.5 °C.

### 2.4. Estimation of Photocatalytic Activity of the (**1**)–(**4**) Oxo-Complexes

**Figure 7:**
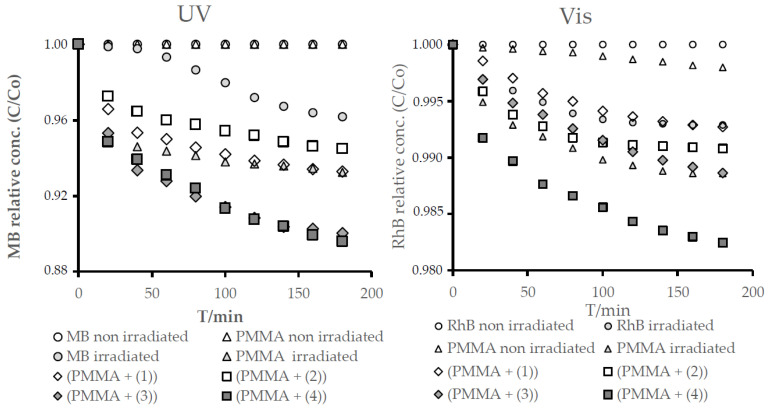
Changes in the concentrations of the methylene blue (MB) and Rhodamine B (RhB) solutions as a function of time for the respective composite materials irradiated with UV and Vis light.

The photocatalytic activity of the (PA + TOCs) systems, with TOCs (**1**)–(**4**), was estimated on the basis of the photodecolorization process of the methylene blue (MB) solution irradiated in the UVA range, according to the ISO 10678:2010 procedure [38,39]. The changes of MB concentrations versus irradiation times for the studied (PMMA + TOCs) composites and the reaction observed rate constants are presented in Figure 7 and Table 5. According to these data, the (PMMA + (**3**)) and (PMMA + (**4**)) composites exhibit the best photocatalytic activity. Simultaneously, in the case of the (PMMA + (**1**)) and (PMMA (**2**)) samples, the very weak photocatalytic activity that was noticed ((PMMA + (**1**)) has activity similar to the pure PMMA (difference in absorbance during 180-min measurements, Δ*A*_180_ = 0.011). The calculated values of Δ*A*_180_ are presented in Table 5. Moreover, the photodegradation of Rhodamine B of the above-mentioned composite films was carried out under VIS light, and the obtained results are presented in Figure 7 and in Table 5 [40].

### 2.5. Antimicrobial Activity of (PMMA + TOCs) Composites

The results of the (PMMA + (**1**)–(**4**)) antimicrobial activity tests against Gram-positive (*S. aureus* ATCC 6538 and *S. aureus* ATCC 25923) and Gram-negative (*E. coli* ATCC 8739 and *E. coli* ATCC 25922) bacteria and yeasts of *Candida albicans* ATCC 10231 of (PMMA + TOCs) composites, where TOCs (**1**)–(**4**) are presented in Table 6. According to these data, all of the (PMMA + TOCs) composites showed high antimicrobial activity against all tested bacteria (*R* between 2.5–6.0). In case of tests performed on the yeasts of *C. albicans* biocidal activity*, R* ≥ 2 or 99% was found for PMMA (**2**)–(**4**)—namely, the complex containing the {Ti_3_O} core and stabilized by the 4-hydroxybenzoic ligand, as well as for complexes with {Ti_4_O_2_} cores but not for PMMA (**1**), which is a complex with a {Ti_3_O} core and stabilized by the -O_2_C-4-PhNH_2_ ligand.

## 3. Discussion

The multinuclear oxo-titanium(IV) complex applicability as a potentially new group of agents with antimicrobial properties was assessed based on carried out investigations. Considering the results of previous works, the oxo-clusters forming the small {Ti_a_O_b_} core structures (i.e., {Ti_4_O_2_} and {Ti_3_O}), and stabilized by -O_2_C-4-PhR’ (R’ = -NH_2_, -OH) ligands have been chosen to further microbiological experiments [20,21,22,23]. The complexes were isolated as micro-grain powders from the reaction mixtures of appropriate titanium alkoxides and organic acids (4:1 molar ratio) at room temperature and in inert atmosphere. The structure of the isolated compounds such as (Ti_3_O(O^i^Pr)_8_(O_2_C-4-PhR’)_2_) (R’ = -NH_2_ (**1**), -OH (**2**)), (Ti_4_O_2_(O^i^Bu)_10_(O_2_C-4-PhR’)_2_) (R’ = -NH_2_ (**3**), and -OH (**4**)) were determined as a result of IR, Raman, and ESI-MS spectra analyses. Studies of UV-Vis-DRS spectra revealed that the absorption maximum of the (**1**)–(**4**) compounds is shifted towards the visible range, i.e., 360–405 nm, with a sharp absorption edge between 480–520 nm (Figure 3). The results of the previous investigations proved that the {Ti_a_O_b_} cores were absorbed in the UV range, which is due to the O2*p*-Ti3*d* charge transfer transition [41]. The shifting of the absorption to the visible range, which was registered in the spectra of the (**1**)–(**4**) compounds, can be explained by the ligand-to-core charge transfer (LCCT) from the -O_2_CR’ (R’ = 4-PhNH_2_ or 4-PhOH) ligands to the tri- or tetranuclear titanium-oxo cores. A similar effect was discussed by Cui et al. for a group of multinuclear Ti(IV)-oxo complexes stabilized by 4-chlorosalicylate ligands [42]. The designated values of the HOMO-LUMO gap energy for the studied oxo-complexes changes in the row (**1**) < (**3**) < (**4**) < (**2**) (Figure 3). The analysis of these data confirms that the type of carboxylate ligand, which stabilizes the {Ti_a_O_b_} skeleton, is the main factor allowed to effectively control the HOMO-LUMO energy gap of multinuclear oxo-complexes. This effect was highlighted during earlier studies of Ti(IV)-oxo clusters of similar core structures and significant different carboxylate ligands [13,14,21,22,41,42]. The influence of the {Ti_a_O_b_} cluster core size on the HOMO-LUMO energy gap (*E*_g_) is definitely more negligible, e.g., the results of Cui et al.’s investigations revealed that, for the {Ti_4_O}, {Ti_11_O_9_}, and {Ti_14_O_12_} clusters stabilized by O^i^Pr and 4-chlorosilicylate ligands, the *E*_g_ values were 3.0, 2.8, and 2.9 eV, i.e., they differed from each other by 0.1 and 0.2 eV [42]. In the case of the studied {Ti_3_O} ((**1**) and (**2**)) and {Ti_4_O_2_} ((**3**) and (**4**)) clusters, these differences amounted to 0.13 and 0.15 eV, respectively (Figure 3).

The research carried out led to the isolation of micro-grained powders of TOCs containing {Ti_3_O} ((**1**) and (**2**)) and {Ti_4_O_2_} ((**3**) and (**4**)} cores, in which the absorption maximum was shifted towards the UVA-Vis range in comparison to TiO_2_ (UV, λ_max_ = 300–350 nm [43]). The type of the carboxylate ligand, which stabilizes the {Ti_a_O_b_} core, influences the HOMO-LUMO gap values (Δ*E*), i.e., Δ*E* (**1**) and (**2**) < Δ*E* (**3**) and (**4**).

Due to the hydrophobic characters of studied powders, their photocatalytic and microbiocidal activity were estimated using (PMMA + TOCs) composite films of 0.25–0.50-μm thickness. The analysis of the SEM images proved that the uniform dispersion of TOC fine grains (3–5 μm) in the PMMA matrix was found only in the (PMMA + (**3**)) composite, while, in the cases of (PMMA + TOCs and TOC = (**1**), (**2**), and (**4**)) composites, the studied materials contained the dispersed, larger (100–300 μm) TOC grains (Figure 4). The nonuniform distribution of TOCs in composite films revealed the analysis of Raman microscopy maps of the (PMMA + (**2**) and (**4**)) films (Figure 5). The large grains of TOCs are separated from each other by the pure polymer sites and by the areas that are formed by fine grains dispersed in PMMA matrices. The thermal characterization of (PMMA + (**1**)–(**4**)) showed a lack of significant differences between their thermal properties and the PMMA matrix. However, higher thermal decomposition temperatures of composite films versus the pure polymer indicate that the Ti(IV)-oxo complex addition to the PMMA matrix improves its thermal stability. A similar effect was observed for the PMMA matrix containing 20% BaTiO_3_ [37]. While analyzing the data presented in Table 3, attention should be paid to the differences in the thermal stability of (PMMA + TOCs) composites resulting from the type of added oxo-cluster. The dispersion of trinuclear oxo-complexes in the PMMA matrix slightly improves the thermal stability of the composite in comparison to the pure polymer, i.e., 0.3–2.7 °C, while this difference is clearer and was found to be 7.6–8.9 °C for the tetranuclear ones (Table 4).

For the reason that studied oxo-complexes can be a potential source of the reactive oxygen species (ROS), before the antimicrobial tests, their photocatalytic activity was determined. Luo et al. [18] and Lv et al. [44] demonstrated the promising photocatalytic activity of the oxo-Ti(IV) clusters dispersed in solutions of MO (methyl orange) and MB. Considering the potential application of TOCs (i.e., (**1**)–(**4**)) clusters, as an antimicrobial agent introduced to the polymer matrix (e.g., PMMA), the photocatalytic activity assessment was made for their (PMMA + (**1**)–(**4**)) composites. Our earlier studies drew our attention to the good photocatalytic activity of (Ti_4_O_2_(O^i^Pr)_10_(O_2_C-4-PhNH_2_)_2_), which the 20 wt.% addition to the polystyrene (PS) matrix or PMMA was one, clearly improved the photodecolorization effect of methylene blue (MB) solution [21,22]. Additionally, trinuclear systems exhibited good photocatalytic activity, which, in the case of (Ti_3_O(O^i^Bu)_8_(O_2_CR’)_2_), (R’ = -C_13_H_9_, -3-PhNO_2_) was proven to be better than for the appropriate tetranuclear oxo-Ti(IV) systems [22]. In the case of the (PMMA + (**1**)–(**4**)) films, the best photocatalytic activity revealed the (PMMA + (**4**)) system irradiated with UVA and Visible light for the MB solution and RhB one, respectively. The PMMA film containing uniformly dispersed fine grains of (**3**) showed slightly weaker activity also in the UVA-Vis range. Cui et al. drew attention to the importance of the size of the oxo-cluster and the related specific surface area [42]. In our case, the cluster size was similar, while the sizes of the grains dispersed in the PMMA matrix were different (Figure 4), which can be associated with the influence of the grain surfaces on their photocatalytic activity. It is interesting that a decrease the of Ti_3_O core size of the oxo-clusters influences the significant decrease of the (PMMA + TOCs) composites (where the TOC (**1**) and (**2**)) have photocatalytic activity, especially in Vis range, and practically no significant changes in UV activity.

The (PMMA + TOCs) systems were sterilized with the use of UVC light, which, according to our UV–Vis-DRS measurements, did not activate the studied samples. In the next stage, the samples were irradiated with natural indoor light directly before inoculation with microorganisms. Considering the results of our earlier works regarding the photocatalytic activity of the (polymer + TOCs) systems [21,22], we assumed that the antimicrobial mechanism of the action of the TOCs could be similar to that of the TiO_2_-assisted ultraviolet treatment (TUV). TUV is a well-known technique used in water disinfection [45,46] or the food industry [47] for the inactivation of microorganisms. The degree of photoinduced deactivation of the microorganisms depends on various treatment parameters, the nature of the microorganisms, and environmental conditions [45,48]. The mechanisms for the bactericidal action of TiO_2_ photocatalysis are associated with the generation of reactive oxygen species (ROSs), such as superoxide radical anion (O_2_^−^·), hydrogen peroxide (H_2_O_2_), and hydroxyl radical (OH·), which cause oxidative damage to living organisms [47,49]. Many authors suggested that the cell membrane is the primary site of ROS attack [50,51,52]. This attack of the cell membrane by ROS induce lipid peroxidation. The cell membrane damage directly leads to the leakage of minerals, proteins, and genetic materials, which is the root cause of cell death [51]. Apart from the cytoplasmic membrane, the supercoiled plasmid DNA, genomic DNA, and internal organelles are destructed when the bacteria are exposed to TUV [47]. Similar mechanisms of PMMA coated with TiO_2_ action against *E. coli* and *S. aureus* were suggested by Su et al. [49]. Moreover, they claimed that a further oxidative attack of internal cellular components accelerates the cell death and, ultimately, results in the decrease of the survival ratio of both tested bacterial strains, namely *S. aureus* and *E. coli* BL21. Furthermore, Pan et al. [53] evaluated the antibacterial properties of nano-trititanate (H_2_Ti_3_O_7_ nanomaterial) against *E. coli* MC1061 and observed that the bactericidal capabilities of these nano-trititanates were more significant compared to nano-TiO_2,_ both with and without exposure to UV light. Various nano-titanates activated with UV light inhibited the bacterial growth in the range of 11.9–57.1%, while these nonirradiated in the range of 25.7–94.7%. The dependence between the photocatalytic properties and antimicrobial activity was also noticed for studied foils of (PMMA + TOCs) composites, containing the 20 wt.% of (**1**)–(**4**) TOCs. The weakest biocidal activity was observed for the (PMMA + (**1**)), which simultaneously showed the worst photocatalytic properties, even irradiated by the light in the UVA-Vis range. The photocatalytic activity of the (PMMA + (**2**)–(**4**)) films was higher, which can explain the strong inhibition of microorganism growth (>99.99%) on their surfaces. It should be noted that the biocidal activity of the (PMMA + TOC) composite films was assessed for 20 wt.% of TOC micro-grain contents due to our earlier studies of (polymer + (**3**)) composite photocatalytic activity (polymer PS and PMMA) [22]. Although ROS generation is the main proposed mechanism of antimicrobial activity of titanium nanomaterials or titanium nanomaterial-containing complexes, the other mechanism of action should be also considered. It is well-known that some nanomaterials, especially metal-based ones, exhibit strong inhibitory effects towards a broad spectrum of bacterial strains. It is claimed that the biocidal effect of these materials, including titanium oxide nanoparticles, results from the “electromagnetic” attraction between the microbe and metal oxide nanoparticles, as microorganisms carry a negative charge while metal oxides carry a positive charge [54]. Overall, metal-based nanoparticles may interact with the sulfur-containing proteins present in the cell envelopes causing irreversible changes in the cell wall structure, resulting in its disruption and affecting the permeability of the cell membrane by altering transport activity through the plasma [55] or disrupting the ATP production [56]. Nanoparticles can further penetrate inside the microbial cells and interact with ribosomes and biomolecules such as proteins, lipids, and DNA, which may alter translation, replication, and other processes in microbial cells. Moreover, the ion release, as a secondary oxidation process, contributes to the biocidal properties of nanomaterials. Metal ions also affect the losses in the ability to replicate DNA, translation process in ribosomes, and protein activities [57,58,59]. They can affect membrane transport and the release of potassium (K^+^) ions from the microbial cells. The increase of membrane permeability caused by both nanomaterials and released ions may lead to the leakage of cellular contents, including ions, proteins, reducing sugars, and, sometimes, cellular energy reservoirs (ATP) [58,60,61,62]. It should, however, be noted that the above-mentioned mechanism can be the result of the direct contact between the nanoparticles and bacterial cell. In the discussed case, the TOC micro-grains were surrounded by the polymer matrix, thus limiting their mutual contact. Therefore, the received results suggested that a mechanism based on the ROS formation seems to be the most probable.

In further investigations, the lowest but still highly active content of TOCs as an antimicrobial agent in the polymer matrix should be determined. Moreover, the possible cytotoxicity of studied (PMMA + TOCs) composites should be assessed, which is extremely important in the case of the potential biomedical applications of these systems. The antimicrobial activity of nanomaterials prevents bacterial adhesion and biofilm formation. This activity is especially important for medical applications of nanomaterials or nanomaterial-containing composites. Biofilms protect the underlying microbes from antibiotics and host defense mechanisms and, thus, may lead to serious infections. Moreover, the antimicrobial properties of nanomaterials may be used to prevent microbial contaminations that cause food spoilage, the spread of foodborne diseases, and bio-fouling of materials [29,63].

## 4. Materials and Methods

### 4.1. Materials

Titanium(IV) isopropoxide (Aldrich, St. Louis, MO, USA), titanium(IV) isobutoxide (Aldrich, St. Louis, MO, USA), 4-aminobenzoic acid (Aldrich, St. Louis, MO, USA), and 4-hydroxybenzoic acid (Aldrich, St. Louis, MO, USA) were purchased commercially and were used without further purification. All solvents used in the synthesis, i.e., tetrahydrofuran (THF), isobutanol (HO^i^Bu), and isopropanol (HO^i^Pr) were distilled before their use and stored in argon atmosphere. The processes of Ti(IV) oxo-complexes synthesis were carried out using the standard Schlenk technique in the inert gas atmosphere (Ar) and at room temperature (RT).

### 4.2. Synthesis of Ti(IV) Oxo-Complexes (TOCs) and (PMMA +TOCs) Composites

The synthesis of [Ti_3_O(O^i^Pr)_8_(O_2_CC_6_H_4_NH_2_)_2_] (**1**): 0.12 g of 4-aminobenzoic acid (0.875 mmol) was added to the solution of 1-mL titanium(IV) isopropoxide (3.5 mmol) in 2 mL of THF/Pr^i^OH (1:1), leading to a clear yellow solution. The solution was left for crystallization. The yield based on acid: 68% (0.31 g). Anal. Calc. for C_38_H_68_O_13_Ti_3_N_2_: C, 50.44; H, 7.52; N, 3.10; Ti, 15.93. Found: C, 50.38; H, 7.48; N, 2.99; Ti, 15.96.

The synthesis of [Ti_3_O(O^i^Pr)_8_(O_2_CC_6_H_4_OH)_2_] (**2**): 0.12 g of 4-hydroxybenzoic acid (0.875 mmol) was added to the solution of 1-mL titanium(IV) isopropoxide (3.5 mmol) in 2 mL of THF/Pr^i^OH (1:1), leading to a clear yellow solution. The solution was left for crystallization. The yield based on acid: 74% (0.33 g). Anal. Calc. for C_38_H_66_O_15_Ti_3_: C, 50.33; H, 7.28; Ti, 15.89. Found: C, 50.81; H, 7.22; Ti, 15.80.

The synthesis of [Ti_4_O_2_(O^i^Bu)_10_(O_2_CC_6_H_4_NH_2_)_2_] (**3**): complex was synthesized, as reported [21]. 0.12 g of 4-aminobenzoic acid (0.875 mmol) was added to the solution of 1.19-mL titanium(IV) isobutoxide (3.5 mmol) in 2 mL of toluene, leading to a clear yellow solution. The solution was left for crystallization. The yield based on acid: 41% (0.22 g). Anal. Calc. for C_54_H_102_O_14_Ti_4_N_2_: C, 52.86; H, 8.38; N, 2.28; Ti, 15.61. Found: C, 53.14; H, 7.83; N, 2.05; Ti, 15.56.

The synthesis of [Ti_4_O_2_(O^i^Bu)_10_(O_2_CC_6_H_4_OH)_2_] (**4**): 0.12 g of 4-hydroxybenzoic acid (0.875 mmol) was added to the solution of 1.19-mL titanium(IV) isobutoxide (3.5 mmol) in 2 mL of THF/Bu^i^OH (1:1), leading to a clear yellow solution. The solution was left for crystallization. The yield based on acid: 63% (0.30 g). Anal. Calc. for C_54_H_100_O_18_Ti_4_: C, 52.77; H, 8.14; Ti, 15.64. Found: C, 51.36; H, 7.92; Ti, 16.01.

The polymer foils containing 20 wt.% of synthesized Ti(IV)oxo-clusters (TOCs) were prepared by an addition of the solution of the TOC solution (ca. 0.25 g of (**1**)–(**4**) TOCs were dispersed in 1 cm^3^ of THF) to the poly(methyl methacrylate) (PMMA) solution (1.0 g of PMMA dissolved in 5 cm^3^ of THF). The resulting mixtures were stirred in an ultrasonic bath for 120 min; in the next step, they were poured into a glass Petri dish and left for the evaporation of the solvent at RT. The composite foil thickness ca. 50 μm were characterized by Raman and IR spectroscopy and scanning electron microscopy.

### 4.3. Analytical Procedures

The structures of the isolated solid reaction products (crystals and powders) were confirmed using vibrational spectroscopy methods, i.e., IR spectrophotometry (Perkin Elmer Spectrum 2000 FTIR spectrophotometer (400–4000 cm^−1^ range, KBr pellets)) and Raman spectroscopy (RamanMicro 200 spectrometer (PerkinElmer, Waltham, MA, USA)). Raman spectra were registered using a laser with the wavelength 785 nm, with a maximum power of 350 mW, in the range 200–3200 cm^−1^, using a 20 × 0.40/FN22 objective lens and an exposure time of 15 s each time. Elemental analyses were performed on an Elemental Analyser vario Macro CHN Elemental Analyser vario Macro CHN Elementar Analysen Systeme GmbH (Elementar, Hanau, Germany). The mass spectra were recorded using the ESI-MS method using a QToFSynapt G2 Si (Waters Corporation, Mundelein, IL, USA) spectrometer. The main measurement parameters: capillary voltage: 2.5–3 kV, source temperature: 110 °C, sampling cone voltage 20–50 V, source offset 40–60 V, desolvation temperature 250–350 °C, cone gas flow: 50.0 dm^3^/h, and desolation gas flow: 798.0–900 dm^3^/h. The produced PMMA + TOCs foil surfaces were studied using a scanning electron microscope with field emission (SEM, Quanta 3D FEG, Houston, TX, USA). Composite materials underwent thermal treatment (Bruker Optik, Ettingen, Germany) in the range 20–500 °C, with the heating speed of 5 °C/min in the atmosphere of nitrogen.

### 4.4. HOMO-LUMO Gap Determination

The HOMO-LUMO gap energy values of isolated (**1**)–(**4**) complexes were determined by using diffuse reflectance UV-Vis spectra (UV-VIS-DRS), which were registered between 200 and 800 nm. Jasco V-750 spectrophotometer was used (JASCO Deutschland GmbH, Pfungstadt, Germany). The recorded spectra were evaluated in terms of energy band gap values via Spectra Manager TM CFR software.

### 4.5. The Photocatalytic Activity Evaluation of (PMMA + TOCs) Composites

The photocatalytic activity of PMMA + TOCs foils (TOCs = (**1**)–(**4**)) was studied by monitoring the degradation processes of MB aqueous solution according to ISO 10678:2010 procedure and, also, Rhodamine B (RhB) solution [38,39,40]. Foil samples of sizes 10 × 10 mm were preconditioned by exposure to UVA or Vis light for 30 h. In the next step, foils were placed in quartz cuvettes with both dye solutions (V = 3.5 cm^3^ and C = 2.0 × 10^−5^ M). After 12 h in the dark, the solutions were replaced by the appropriate test of MB and RhB solutions (for both: V = 3.5 cm^3^ and C = 1.0 × 10^−5^ M). The prepared samples were exposed to UVA irradiation (18-W lamp, 340–410-nm range, with a maximum at 365 nm) and Vis light (77-W tungsten halogen lamp, range of 350–2200 nm). All cuvettes were covered with quartz glass panes during irradiation. MB absorbance at 660 nm and RhB absorbance at 554 nm were registered (Metertech SP-830 PLUS, Metertech, Inc., Taipei, Taiwan) after 0, 20, 40, 60, 80, 100, 120, 140, 160, and 180 min of irradiation. Percentage of MB decolorization was calculated using the equation

% dye decolorization = ((*C*_0_ − *C_t_*)/*C*_0_) × 100 = ((*A*_0_ − *A_t_*)/*A*_0_) × 100 (1)

where *C*_0_ is an initial concentration of dye, *C_t_* is a dye concentration at a given time *t*, and *A*_0_ and *A_t_* are absorbances at 0 and *t* times [38,64].

The rate of both photodegradation processes was estimated by a simple calculation of a difference in absorbance during 180-min measurements (Δ*A*_180_). Such kind of calculation is a result of a low degradation degree of dyes.

### 4.6. The Evaluation of Antimicrobial Properties of (PMMA + TOCs) Systems

Antimicrobial activity of PMMA + TOCs (TOCs = (**1**)–(**4**)) composite foils (30 × 30 × 0.05 mm) was studied against Gram-positive (*S. aureus* ATCC 6538 and *S. aureus* ATCC 25923) and Gram-negative (*E*. *coli* ATCC 8739 and *E. coli* ATCC 25922) bacteria and yeasts of *Candida albicans* ATCC 10231 using method according to the ISO 22196:2011 standard [65]. All strains were purchased from the American Type Culture Collection (Manassas, VA, USA). PMMA +TOC specimens were sterilized using a UVC lamp for 30 min in the laminar hood (Bioquell, Hampshire, UK) and placed in sterile Petri plates. The earlier investigations revealed that the absorption maximum of the (PMMA + TOCs) samples was found at the UVA-Vis border, which enabled the use of UVC radiation in order for their sterilization. Microbial suspension (1.0–1.8 × 10^6^ colony-forming units (cfu) cm^3^/^−1^) prepared in sterile deionized water was placed on the surfaces of the appropriate PMMA + TOCs samples and covered with sterile foil films (polypropylene; PP) and incubated for 24 h at 37 °C in a humid atmosphere. The PP film was then removed and microbial suspension collected into a 2-cm^3^ centrifuge tube. Subsequently, serial ten-fold dilutions of each sample were prepared. Aliquots (100 µL) of each dilution were aseptically spread over the surface of Tryptic Soy Agar (TSA, Becton Dickinson, Sparks Glencoe, MD, USA) or Sabouraud Dextrose Agar (SDA, Becton Dickinson) in Petri plates for the bacteria and fungi, respectively. Inoculated samples were incubated at 37 °C for 24 h. Assays were performed in triplicate. After incubation, colony-forming units (cfu) were counted on the agar plates. Control was PMMA inoculated with the test microorganism.

The antimicrobial activity was calculated using Formula (2), according to the ISO 22196:2011 standard [65]:

𝑅 = *U_t_* − *A_t_* (2)

where *U_t_* is the average of the common logarithm of the number of viable bacteria in cells/cm^2^ recovered from the untreated test specimens (PMMA) after 24 h, and *A_t_* is the average of the common logarithm of the number of viable bacteria in cells/cm^2^ recovered from the treated test specimens (PMMA + TOCs) after 24 h. *R* ≥ 2 determines the biocidal activity

The percentage reduction (*R*%) of the bacterial or fungal growth was calculated using Formula (3):

𝑅% = ((𝐵 − 𝐴)/B) × 100(3)

where 𝑅 is the biocidal rate (%), 𝐵—the average number of microorganisms on unmodified PMMA in T_0_, and 𝐴—the average number of microorganisms on the surfaces of the studied (PMMA + TOCs) composites after 24 h. The 100-times reduction of the number of colonies determines at least 99% of the reduction of microbial growth (biocidal activity).

## 5. Conclusions

The work carried out led to the production of oxo-titanium(IV) complexes (TOCs), i.e., (Ti_3_O(O^i^Pr)_8_(O_2_C-4-PhR’)_2_) (**1**) and (**2**) and (Ti_4_O_2_(O^i^Bu)_10_(O_2_C-4-PhR’) (**3**) and (**4**) (R’ = -NH_2_ and -OH), which structures were confirmed by IR, Raman spectroscopy, and ESI-MS spectrometry. The dispersion of 20 wt.% of TOCs (**1**)–(**4**) in the PMMA matrix allowed the formation of (PMMA + TOCs) composite films for which the thermal and photocatalytic properties and biocidal activity were estimated. The obtained results suggest that (Ti_4_O_2_(O^i^Bu)_10_(O_2_C-4-PhOH)_2_) (**4**) can be used for the production of composites (polymer + TOC), which, in the form of films, coatings, or resin additives, can be used as a bactericidal agent in various areas of our lives. It should be noted that the potential use of materials based on PMMA with the addition of TOCs in dentistry would require medical experiments carried out in accordance with the procedures specified by Isola et al. [66,67].

## Figures and Tables

**Table 1:** The compositions of the reaction mixtures. THF: tetrahydrofuran.

4:1 Molar Ratio		Isolated	
Ti(OR)_4_	HOOCR’	Solid Product	Solvent
Ti(O^i^Pr)_4_	HOOC-4-PhNH_2_	(1)	THF/^i^PrOH
Ti(O^i^Pr)_4_	HOOC-4-PhOH	(2)	THF/^i^PrOH
Ti(O^i^Bu)_4_	HOOC-4-PhNH_2_	(3)	Toluene
Ti(O^i^Bu)_4_	HOOC-4-PhOH	(4)	THF/^i^BuOH

**Table 2:** Results of the vibrational spectra studies of the (**1**)–(**3**) complexes (the band intensity: strong (s), middle (m), weak (w), very weak (vw).

	(1)		(2)		(3)		(4)	
Modes	IR	R	IR	R	IR	R	IR	R
ν(OH) ν(NH_2_)	3200–3450	3162 (w)	3300–3500		3200–3450	3175 (w)	3300–3500	
ν(CC) (Ph)	1622 (m) 1601 (m)	1603 (s)	1622 (m) 1603 (m)	1595 (s)	1621 (m) 1603 (m)	1602 (s)	1622 (m) 1603 (m)	1594 (s)
δ(NH_2_)	1587 (m)				1589 (m)			
ν_as_(COO)	1533 (m) 1516 (m)	1524 (m)	1536 (m) 1516 (m)	1519 (w)	1523 (m)	1524 (m)	1536 (m) 1510 (w)	1518 (w)
ν_s_(COO)	1462 (w)	1436 (m)	1464 (w)	1450 (w)	1497 (m)	1459 (w)	1498 (m)	1445 (w)
δ(NH_2_)	1362 (m)	1306 (m)			1299 (m)	1300 (m)		
ν(C-O) + δ(CC)	1014 (s)		1016 (m)		1036 (m)		1016 (m)	
ν(Ti-OR) + ν(CC) + δ(CH)	986 (m) 949 (m)	856 (m)	988 (m) 951 (m)	838 (m)	971 (m) 948 (w)	856 (m)	988 (m) 951 (w)	838 (m)
ν(CH) 1,4-Ph	780 (m)		782 (m)		784 (s)		782 (m)	
ν(Ti_3_-(μ_3_-O)	750 (w) 728 (w)	747 (vw) 719 (w)	755 (vw) 727 (vw)	748 (vw)				
ν(Ti_2_-(μ-O)					703 (m)	690 (vw)	702 (m)	687 (vw)
ν(Ti_4_-(μ_4_-O)					639 (m)	623 (w)	634 (m)	610 (vw)
δ(CCC)	683 (w)	622 (m)	701 (w)	678 (m)				
	666 (m)	605 (w)	638 (m)	638 (w)				
	622 (m)		629 (w)					
ν(Ti_3_-(μ_3_-O)	561 (w)		564 (w)	539 (vw)				
ν(Ti_4_-(μ_4_-O)					532 (m)	548 (vw)	556 (m)	536 (vw)
			532 (w)	510 (w)				
ν(Ti_3_-(μ_3_-O)	486 (m)		483 (m)					
		417 (w)		419 (w)				
ν(Ti_3_-(μ_3_-O)		342 (vw)		350 (vw)				

**Table 3:** The results of electrospray ionization-mass spectrometry (ESI-MS) spectra studies of the (**1**)–(**4**) complexes. TOCs: tetranuclear oxo-titanium(IV) complexes.

TOCs	*m*/*z*	Fragmentation Ion	Intensity (%)
[Ti_3_O(O^i^Pr)_8_(O_2_C-4-PhNH_2_)_2_] (**1**)	768	(Ti_3_O(O^i^Pr)_8_(O_2_C-4-PhNH_2_))^+^	8
	550	(Ti_3_O(O^i^Pr)_2_(O_2_C-4-PhNH_2_))^+^	36
[Ti_3_O(O^i^Pr)_8_(O_2_C-4-PhOH)_2_] (**2**)	847	(Ti_3_O(O^i^Pr)_7_(O_2_C-4-PhOH)_2_)^+^	26
	769	(Ti_3_O(O^i^Pr)_8_(O_2_C-4-PhOH))^+^	90
[Ti_4_O_2_(O^i^Bu)_10_(O_2_C-4-PhNH_2_)_2_] (**3**)	1080	(Ti_4_O_2_(O^i^Bu)_8_(O_2_C-4-PhNH_2_)_2_)^+^	5
	881	(Ti_4_O_2_(O^i^Bu)_9_)^+^	5
[Ti_4_O_2_(O^i^Bu)_10_(O_2_C-4-PhOH)_2_] (**4**)	1229	(Ti_4_O_2_(O^i^Bu)_10_(O_2_CPhOH)_2_) + H^+^	8
	945	(Ti_4_O_2_(O^i^Bu)_8_(O_2_CPhOH))^+^	10

**Table 4:** Thermal parameters received from thermogravimetric analysis (TGA) and differential scanning calorimetry (DSC) of the poly(methyl methacrylate) (PMMA) and (PMMA + TOCs) composites (T_g_ = glass transition temperature, T = evaporation temperature of an unreacted monomer, T_m_ = melting temperature. T_max_ = temperature in the thermal transition maximum, Δm = thermal transition weight loss).

	DSC			TGA		
Composite	T_g_/°C	T/°C	T_m_/°C	Stage I T_max_/°C/Δm/%	Stage II T_max_/°C/Δm/%	Solid Residue at 450 °C (%)
PMMA	99.6	150.8	368.6	194.9/12	365.1/85	3
(PMMA+(1))	100.6	-	370.4	188.2/12	367.8/70	18
(PMMA+(2))	101.2	-	375.7	183.9/11	365.4/74	15
(PMMA+(3))	99.0	-	377.8	159.9/15	372.7/76	9
(PMMA+(4))	115.2	-	364.5	171.0/10	374.0/79	11

**Table 5:** Organic dye decolorization percentages and Δ*A*_180_ parameters for the studied reactions in relation to the composites.

**Composite**	**MB Decolorization ^a^ (%)**	**Δ*A*_180_**	**Δ*A*_180_** **in Reference to PMMA**
PMMA+(1)	6.70	0.067	–0.001
PMMA+(2)	5.50	0.055	–0.013
PMMA+(3)	9.97	0.100	0.032
PMMA+(4)	10.42	0.104	0.036
PMMA(irradiated)	6.76	0.068	-
**Composite**	**RhB Decolorization ^b^ (%)**	**Δ*A*_180_**	**Δ*A*_180_** **in Reference to PMMA**
PMMA+(1)	0.73	0.007	–0.004
PMMA+(2)	0.92	0.009	–0.002
PMMA+(3)	1.14	0.011	0
PMMA+(4)	1.75	0.018	0.007
PMMA(irradiated)	1.14	0.011	-

^a^ Methylene blue (MB) decolorization at the end of the measurements (t = 180 min). ^b^ Rhodamine B (RhB) decolorization at the end of the measurements (t = 180 min).

**Table 6:** Antimicrobial activity of the (PMMA +TOCs) systems (*R =* biocidal rate (%), *R*% = reduction percentage).

Microorganisms	PMMA+ (1)		PMMA+ (2)		PMMA+ (3)		PMMA+(4)	
	*R*	*R*%	*R*	*R*%	*R*	*R*%	*R*	*R*%
*E*. *coli* ATCC 8739	2.5	99.6	6.0	>99.99	6.0	>99.99	6.0	>99.99
*E*. *coli* ATCC 25922	3.3	99.95	6.0	>99.99	6.0	>99.99	6.0	>99.99
*S. aureus* ATCC 6538	6.0	>99.99	6.0	>99.99	6.0	>99.99	6.0	>99.99
*S. aureus* ATCC 25923	6.0	>99.99	6.0	>99.99	6.0	>99.99	6.0	>99.99
*C. albicans* ATCC 10231	1.7	85.0	6.0	>99.99	6.0	>99.99	6.0	>99.99

*R* ≥ 2 is a biocidal effect when the microbial growth is reduced at least 100 times (99.0%).
